# Post-traumatic skin and soft tissue infection due to *Aeromonas hydrophila*

**DOI:** 10.4103/0972-5229.78228

**Published:** 2011

**Authors:** Bijayini Behera, Sandeep Bhoriwal, Purva Mathur, Sushma Sagar, Maneesh Singhal, M. C. Misra

**Affiliations:** **From:**Department of Microbiology, JPNA Trauma Centre, All India Institute of Medical Sciences, New Delhi, India; 1 Department of Orthopedics, JPNA Trauma Centre, All India Institute of Medical Sciences, New Delhi, India; 2 Department of Laboratory Medicine, JPNA Trauma Centre, All India Institute of Medical Sciences, New Delhi, India; 3 Department of General Surgery, JPNA Trauma Centre, All India Institute of Medical Sciences, New Delhi, India; 4 Department of Surgical Disciplines and Chief, JPNA Trauma Centre, All India Institute of Medical Sciences, New Delhi, India

**Keywords:** *Aeromonas hydrophila*, skin and soft tissue infection, trauma

## Abstract

We report a case of posttraumatic skin and soft tissue infection in a patient who sustained laceration after being hit by a water tanker. *Aeromonas hydrophila* was isolated from pus and was identified to the species level by Vitek 2 and a battery of biochemical tests. The patient responded to thorough drainage, debridement of wound and 2 weeks of intravenous antibiotics. The patient was taken up for split skin grafting of the raw area. She was discharged with satisfactory graft uptake after 1 week without any further antibiotics advice. Follow-up after 3 weeks was satisfactory with healthy cover on the raw area and normal weight bearing on the left leg.

## Introduction

The genus *Aeromonas* is a member of the family Vibrionaceae. *Aeromonas hydrophila* is the most commonly isolated species associated with human infections.[1] Human infections caused by the *Aeromonas* species are rare and include gastrointestinal illness, soft tissue infections, pneumonia, meningitis, endocarditis, osteomyelitis, and septic arthritis.[1] Skin and Soft tissue and infections are second in prevalence only to gastrointestinal illnesses in these patients.[1] Herein; we describe a case of post-traumatic skin and soft tissue infections due to A. *hydrophila* in an otherwise immunocompetent individual.

## Case Report

A 40 yearold female presented to the emergency department of our level 1 trauma center, 1.5 hours after being hit by a water tanker. The patient had sustained a lacerated wound over medial aspect of left leg, extending from knee to lower one-third of leg with no underlying bone injury. After initial assessment and management, thorough cleansing of the wound, was done followed by suturing with nylon and she was discharged from the emergency department. She was advised to take oral amoxicillin and analgesics for 3 days and was called for follow-up after 3 days. Three days later, the patient presented to follow-up out-patient department with fever and pain in the left leg. The leg was swollen with erythematous changes along with purulent discharge from the wound. The patient was admitted, and the sutures were opened.

On evaluation, her past medical history was unremarkable. Vital signs revealed an oral temperature of 101.6°F, a pulse rate of 78/minute, a blood pressure level of 130/80 mm Hg, and a respiration rate of 22/minute. Physical examination of the left lower extremity revealed a l9-cm laceration overlying the medial aspect of left leg, extending from knee to lower one-third of leg, accompanied by diffuse edema and erythema extending proximally to the posterior medial thigh. The left inguinal and popliteal lymph nodes were enlarged and painful. A preoperative diagnosis of de-gloving injury of left leg with abscess was made. A thorough drainage and debridement was performed under general anesthesia; about 100 ml of foul smelling blood streaked purulent fluid was drained and intra-operative aerobic and anaerobic cultures were obtained. The patient was put on IV antibiotics (amoxicillin/clavulanic acid, amikacin, and metronidazole) in the ward. Gram stain of the purulent aspirate demonstrated many white blood cells and few Gram-negative bacilli. After overnight incubation, large, round, opaque β-hemolytic colonies were obtained on blood agar. The colonies were non-lactose fermenting on MacConkey agar. The Gram-negative rod was identified as *Aeromonas hydrophila* by the Vitek 2 system. A battery of biochemical tests was performed to identify the organism to the species level. The Gram-negative rod was oxidase positive and gelatinase negative. The organism was positive for lysine decarboxylation (Moeller), arginine dihydrolase (Moeller), and the Voges-Proskauer test. The results of biochemical tests are summarized in Table 1. The organism was sensitive to amikacin, cefepime, cefixime, cefoperazone/sulbactam, ceftazidime, ceftriaxone, chloramphenicol, ciprofloxacin, gentamicin, imipenem, levofloxacin, meropenem, netilmicin, tetracycline, ticarcillin, trimethoprim/sulfamethoxazole, and tobramycin, and was resistant to ampicillin and piperacillin. The antimicrobial susceptibility was done by the Vitek 2 advanced expert system (BioMerieux) The antibiotic therapy was changed to intravenous piperacillin and tazobactum with netilimicin for 2 weeks after the anitibiogram profile was obtained. Daily dressing of the wound was done. Slight blackish discoloration of the skin was noted in the immediate postoperative period, which gradually subsided in 3-4 days time. The patient remained afebrile and the wound also became clean with healthy granulation tissue. The subsequent wound swab cultures taken after 7 days were negative. As the hemoglobin of the patient was low, she could be taken up for split skin grafting of the raw area of the left leg nearly after 3 weeks. She was discharged with satisfactory graft uptake after 1 week without any further antibiotics advice. Follow-up after 3 weeks was satisfactory with healthy cover on the raw area and normal weight bearing on the left leg.

**Table 1:** Biochemical reactions of the isolate

Test	Results
Motility	Motile
Hugh and Leifson’s O/F test	Fermentative
Indole production	+
Citrate (Simmon’s utilization)	−
H_2_S production (Triple Sugar Iron Agar)	−
Urea (Christensen)	−
Nitrate production	+
Lysine decarboxylase	+
Arginine dihydrolase	+
Ornithine decarboxylase	−
Gelatin hydrolysis at 22°C	+
Growth in KCN	+
Acid production from	
Glucose	+
Lactose	−
Maltose	−
Sucrose	−
Xylose	−
Growth at	
35°C	+
37°C	+
42°C	−

## Discussion

Soft tissue infections caused by *A. hydrophila* typically occur after soft tissue trauma due to exposure to water or contaminated objects in water.[2] Soft tissue destruction occurs within 72 hours because of the myonecrosis associated with this organism.[3] Causes of injury range from puncture wounds to abrasions.[3] The severity of these soft tissue infections depends on host defense factors. Septicemia and bacteremia are usually seen in the immunocompromised patient.[1] Fatal myofascial necrosis, gas gangrene, abscesses, and extremity amputation have all been reported from infections caused by *A. hydrophila.*[1]

The rapid onset of cellulitis in the setting of soft tissue trauma and exposure to water should alert the clinician regarding the possibility of infection with this organism.[4] Treatment of soft tissue infections caused by A. *hydrophila* involves both medical and surgical regimens.[5] Incision and drainage is required for puncture wounds and for other soft tissue infections.[5] After surgical decompression, parenteral and oral antibiosis is an essential part of the treatment. While clinical isolates of *Aeromonas* are susceptible to a wide range of antibiotics, they are universally resistant to penicillin, ampicillin, carbenicillin, and cefazolin.[1] The microbiology laboratory should be alerted to the clinical setting so that an oxidase or deoxyribonuclease screen is performed to differentiate *A. hydrophila* from microbiologically similar organisms and, hence, avoid the possibility of a fulminant infection. Similarly, appropriate antimicrobial therapy, as determined by culture and sensitivity report, is of paramount importance in addition to early surgical exploration of wounds, for optimal management of these rare, but rapidly progressive, infections.
